# Prosthesis-Based 3D Guide System for Maxillary Implant Placement: A Feasibility Study Using a Split-Mouth Evaluation

**DOI:** 10.3390/dj14070395

**Published:** 2026-07-01

**Authors:** Marco Tudts, Tashia Moodley, Rani D’haese, Stefan Vandeweghe

**Affiliations:** 1 Department of Periodontology, Dental School, Faculty of Medicine and Health Sciences, Ghent University, 9000 Ghent, Belgium; 2 Cosmident, Private Practice, 2500 Lier, Belgium; moodleytashia@gmail.com; 3 Department of Reconstructive Dentistry, Dental School, Faculty of Medicine and Health Sciences, Ghent University, 9000 Ghent, Belgium; rani.dhaese@ugent.be (R.D.); stefan.vandeweghe@ugent.be (S.V.)

**Keywords:** guided implant surgery, split-mouth design, full-rough implant, hybrid implant surface, marginal bone loss, OHIP-14, edentulous maxilla, prosthesis-based guide

## Abstract

**Background/Objectives:** To evaluate the clinical feasibility and short-term radiographic outcomes of adapting a prosthesis-based guide system originally developed for single-implant placement for multi-implant placement in the edentulous maxilla, and to compare implant-level marginal bone change from prosthetic loading to one year between full-rough (IBT/IBNT/IBR) and hybrid-surface (MSC-IBT/MSC-IBNT) implants in a split-mouth design. Patient-reported outcomes were assessed with the OHIP-14 questionnaire. **Methods:** Fifteen patients with an edentulous maxilla received four or five maxillary implants placed flaplessly using a 3D-printed prosthesis-based guide incorporating polyether-ether-ketone (PEEK) rails and interchangeable angulation-correction sleeves (0°, 12°, 24°). Implants had either a fully roughened or a hybrid (rough apical and middle third with a machined coronal collar) surface. Side allocation was non-randomized: the hybrid side was assigned by alternating sequence and three patients received only full-rough implants for prosthetic reasons. All patients followed a delayed loading protocol and received a screw-retained zirconia fixed bridge. Standardized periapical radiographs were obtained at definitive prosthetic loading (baseline) and at the 12-month follow-up. Implant-level marginal bone loss was calculated as the mean of mesial and distal measurements per implant. All radiographic measurements were performed by two independent examiners (M.T. and T.M.); intra-rater reliability (M.T., remeasurement of 10 radiographs) and inter-rater reliability (M.T. versus T.M., full dataset) were quantified by intraclass correlation coefficient (ICC; two-way mixed effects, absolute-agreement, single-measurement). The primary split-mouth surface comparison was performed at the patient level by averaging implant-level change scores within each surface for each patient and comparing the paired patient-level means with a Wilcoxon signed-rank test. No a priori sample-size calculation was performed; the study was designed as a feasibility pilot. **Results:** Sixty-one implants were placed in 15 patients (seven male, eight female; mean age 62.5 ± 8.9 years; three current smokers). Four implants in three patients required removal and replacement during the observation period (three early failures, one late failure; per-implant early-failure rate of 3/61, 4.9%); one patient (P7) withdrew from clinical follow-up. Paired baseline and 12-month radiographs were available for 53 implants from 14 patients. Median implant-level marginal bone level increased from 0.38 mm (IQR 0.20–0.54) at baseline to 0.78 mm (IQR 0.47–1.32) at 12 months (paired Wilcoxon signed-rank, *p* < 0.001); two implants exceeded 4 mm of bone change at 12 months. In the patient-level paired surface comparison (*n* = 8 patients contributing at least one full-rough and one hybrid implant with paired data), full-rough implants showed less 12-month marginal bone change than hybrid implants in every paired patient (median paired difference full-rough hybrid of −0.49 mm; Hodges–Lehmann pseudo-median of 0.55 mm; paired Wilcoxon *p* = 0.012). OHIP-14 scores at one year (*n* = 14) showed a pronounced floor effect, with most patients scoring zero across most domains. Both intra-rater (M.T.) and inter-rater (M.T. versus T.M.) reliability showed good agreement (ICC = 0.85). **Conclusions:** A prosthesis-based guide system originally validated for single-implant placement can be feasibly adapted for flapless multi-implant rehabilitation of the edentulous maxilla, with early clinical and radiographic outcomes broadly consistent with comparable published series. Contrary to the design rationale that a machined coronal collar would limit early crestal remodeling, full-rough implants showed less 12-month within-patient bone change than hybrid implants in the eight paired patients; this finding is preliminary and hypothesis generating given the small, unbalanced paired sample and the contrast with larger published series. The approach is best characterized as a reduced infrastructure alternative to proprietary guided-surgery platforms, remains operator dependent, and requires confirmation in formally powered, balanced split-mouth trials with concealed allocation, placement anchored bone level measurement, postoperative CBCT for deviation quantification, and longer follow-up.

## 1. Introduction

Despite the increase in oral healthcare and the availability of various prosthetic solutions, edentulism remains a widespread condition and its prevalence continues to increase [1]. For those patients, dental implants offer an alternative to the complete removable denture which often present problems with retention, reduced chewing efficiency, mucosal irritation and speech problems [2,3,4]. However, implant placement in the edentulous maxilla can be challenging due to its low bone density and sinus pneumatization [5,6,7]. Conventional implant-retained maxillary prostheses are typically supported by multiple implants connected through splinted frameworks to optimize load distribution. Although these protocols provide predictable outcomes, they are resource intensive, rely on laboratory infrastructure, and may be financially inaccessible for many patients [8,9,10]. Simplified overdenture concepts that have demonstrated success in the mandible are less readily adaptable to the maxilla because of inherent anatomical and biomechanical limitations [10,11].

Precise three-dimensional implant planning and placement are critical in the maxilla, where thin cortical plates, variable trabecular density and proximity to the nasal cavity and maxillary sinus increase the risk of surgical complications [12,13]. Guided surgical systems assist clinicians by translating prosthetic planning into accurate implant trajectories, helping to minimize sinus or nasal cavity encroachment, labial plate perforation and unfavorable angulation [14,15]. These systems are particularly beneficial in flapless or minimally invasive approaches. In contrast, freehand placement is associated with greater angular deviation, positional inaccuracy and higher risk of compromised prosthetic alignment [16,17,18].

Although fully digital guided-surgery platforms enhance accuracy, their cost, software dependency, time and reliance on specialized laboratory workflows can limit their use in many clinical settings [12,14]. To address these limitations, a prosthesis-based guide system was developed for single-implant rehabilitation and validated in vitro for placement in the mandible [19] and was here adapted for multi-implant placement in the edentulous maxilla. Prosthesis-based guidance using duplicated denture and stereolithographic designs has been described previously [14,20,21]; the distinguishing features of the present system are (i) repositionable polyether-ether-ketone (PEEK) rails embedded in the duplicated denture allowing the bucco-palatal position of each sleeve to be adjusted intraoperatively without re-printing the guide, (ii) interchangeable angulation correction sleeves (0°, 12°, and 24°) that permit trajectory adjustment within a single guide and (iii) application of this modular concept to multi-implant maxillary rehabilitation rather than to the single-implant mandibular use case. The workflow still requires CBCT imaging, prosthetically driven planning and access to a desktop 3D printer and is therefore best characterized as a reduced infrastructure alternative to proprietary guided-surgery platforms rather than as low infrastructure in absolute terms [20,21]. Long-term implant success depends not only on appropriate planning but also on effective plaque control and regular supportive maintenance [22,23]. Roughened implant surfaces have been shown to enhance bone-to-implant contact and accelerate osseointegration; however, once exposed to the oral environment they may facilitate biofilm accumulation and the progression of peri-implant inflammation [24,25].

Hybrid-surface implants combining a roughened apical and middle third with a machined coronal collar were introduced to preserve the benefits of osseointegration while potentially reducing crestal plaque retention and peri-implant tissue breakdown during early bone remodeling [26,27]. Nevertheless, clinical evidence directly comparing hybrid and fully rough implant surfaces within the same maxillary overdenture protocol remains limited [28,29]. A split-mouth design allows intra-patient comparison under identical anatomical, surgical and prosthetic conditions thereby reducing inter-subject variability and increasing the internal validity of surface-related outcome assessment [30].

The present pilot study therefore evaluated the feasibility of adapting a prosthesis-based guide system for flapless multi-implant placement in the edentulous maxilla. The primary objective was to compare implant-level marginal bone change between full-rough and hybrid-surface implants from prosthetic loading to one year in a split-mouth design. Secondary objectives were to assess the clinical feasibility of the adapted guide system and one-year patient-reported outcomes using the OHIP-14 questionnaire.

## 2. Materials and Methods

### 2.1. Study Design and Ethical Approval

This prospective pilot clinical study evaluated a prosthesis-based guided-surgery concept for implant rehabilitation in edentulous patients. A previously developed single-implant guide system was adapted for flapless, multi-implant placement in the maxilla using a prosthesis-based three-dimensional (3D) printed guide [21]. The study was designed as a feasibility pilot and no a priori sample-size calculation was performed.

The study was approved by the Ethics Committee of the University of Ghent, Belgium (EC-BE670201940408, approval date: 19 June 2019), and conducted in accordance with the Declaration of Helsinki and Good Clinical Practice (GCP) guidelines. Written informed consent was obtained from all participants prior to enrolment.

### 2.2. Patient Selection

Fifteen patients with an edentulous maxilla were consecutively enrolled between 2020 and 2022. Eligible participants were adults with complete maxillary edentulism and sufficient bone volume to accommodate four implants, as confirmed by cone-beam computed tomography (CBCT). The condition of the opposing mandibular arch was documented and classified as dentate, edentulous with a removable complete denture or edentulous with an implant-supported overdenture as this factor may influence prosthetic loading conditions [5].

Exclusion criteria included active oral infections, uncontrolled systemic disease, immunosuppression, a history of head-and-neck radiotherapy, chemotherapy or bisphosphonate therapy and any condition that could compromise wound healing or osseointegration. Smoking status was recorded at baseline as a potential confounding variable.

### 2.3. Preoperative Planning and Guide Fabrication

At the initial visit each patient underwent CBCT imaging to enable prosthetically driven digital planning of implant trajectories. A duplicate of the patient’s existing maxillary denture was digitally designed and three-dimensionally printed to serve as a prosthesis-based surgical guide. Four polyether-ether-ketone (PEEK) rails were incorporated into the duplicate prosthesis, each housing an adjustable PEEK guide sleeve. Special drill keys were fitted in the PEEK, which guided drill sequences smoothly without the risk of making a hole in the PEEK sleeve wider and also ensured a more accurate drill guidance. The guide sleeves could be repositioned bucco-palatally within the rails to optimize the planned insertion path and accommodate minor discrepancies between virtual planning and clinical conditions. Interchangeable angulation-correction sleeves (0°, 12°, and 24°) allowed further adjustment of implant trajectory without reprinting the entire guide. Polyether-ether-ketone was selected for the rails and sleeves because it combines adequate mechanical rigidity for drill guidance with biocompatibility, radiolucency on the verification CBCT, and a cost and machinability profile compatible with in-house 3D printing. Metal sleeves were considered but were judged less suitable in this reduced-infrastructure setting because of cost, separate sourcing, and the image artifacts they introduce on intraoperative imaging.

**Figure 1:**
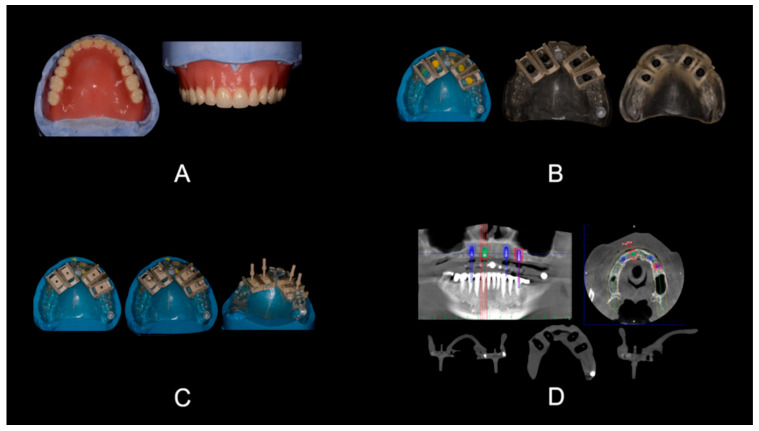
Preoperative planning and fabrication of a prosthesis-based surgical guide system for maxillary implant placement. (**A**) Original complete denture used as the basis for guide design; (**B**) duplicate denture with embedded polyether-ether-ketone (PEEK) rails to enable repositioning of the guide sleeve; (**C**) duplicate denture with directional indicators and adjustable guide sleeve showing angulation options (0°, 12°, and 24°); (**D**) 3D planning based on cone-beam computed tomography (CBCT) with radiopaque fiduciary markers, enabling prosthetically driven implant trajectory selection.

CBCT imaging was used for preoperative planning and to verify correct intraoral seating and spatial alignment of the duplicated prosthesis with integrated PEEK rails. The workflow and design features of the prosthesis-based guide, including PEEK rail integration and adjustable angulation sleeves, are illustrated in Figure 1. This digital pre-surgical planning protocol has been described in vitro by Tudts et al. [19] for single implant placement in the mandible.

### 2.4. Surgical Procedure

**Figure 2:**
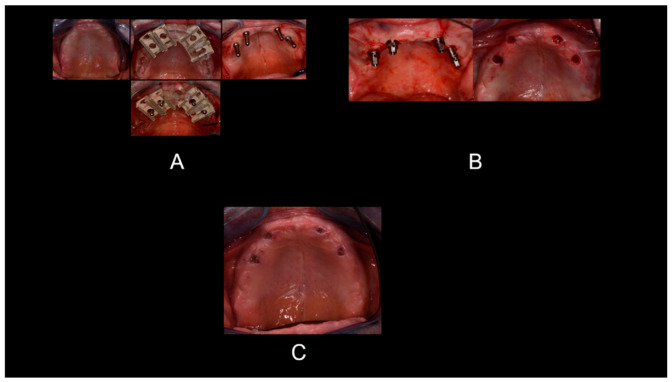
Intraoperative workflow for flapless maxillary implant placement. (**A**) Intraoral seating of the modified denture-based surgical guide prior to osteotomy, ensuring stable mucosal support and accurate positioning; (**B**) osteotomy preparation through the polyether-ether-ketone (PEEK) guide sleeve, enabling controlled angulation and depth; (**C**) fixture placement using a flapless approach with the guide system in place, preserving soft tissue architecture and minimizing surgical morbidity.

All maxillary implant placements were performed under local anesthesia using a flapless, mucosa-supported approach. The prosthesis-based surgical guide was positioned intraorally and verified for complete seating and stability prior to osteotomy preparation. The intraoperative sequence is illustrated in Figure 2.

Osteotomies were prepared through the integrated PEEK guide sleeves following the manufacturer’s drilling protocol. Four implants were placed sequentially in predetermined anterior and premolar positions according to the planned angulation and depth. In one patient, an additional implant was placed (*n* = 5) due to anatomical and prosthetic requirements. Primary stability was verified clinically, after which the guide was removed and healing abutments were attached. No flap elevation or suturing was required. Implant diameters, lengths and surface characteristics are detailed in Table 1. In accordance with the manufacturer’s nomenclature, IBT denotes implants of 4.0 mm diameter, IBNT implants of 3.25 mm diameter, and IBR a 4.0 mm full-rough variant. Full-rough implants (IBT, IBNT, IBR) presented a moderately rough surface along their entire length whereas hybrid implants (MSC-IBT, MSC-IBNT) featured a roughened apical and middle third with a machined coronal collar.

### 2.5. Side Allocation and Surface Assignment

The split-mouth configuration assigned full-rough implants to one side of the arch and hybrid implants to the contralateral side. Side allocation was not formally randomized: the side receiving hybrid implants was assigned by alternating sequence across the enrolment list, without concealed allocation. Three patients (P1, P4, P10) received full-rough implants on both sides for prosthetic reasons; inter-implant spacing on the contralateral side did not allow a hybrid placement within the planned prosthetic envelope (4 full-rough/0 hybrid). One patient (P7) received a 1 full-rough/3 hybrid allocation rather than a 2/2 split-side configuration; this is identified as a protocol deviation. The remaining 11 patients received the planned 2/2 (or 3/2) split-mouth allocation. The per-patient distribution of full-rough and hybrid implants is given in Table 1.

### 2.6. Postoperative Care and Healing

Patients received antibiotic prophylaxis with amoxicillin 1 g twice daily for four days or clindamycin 300 mg three times daily in cases of penicillin allergy. Analgesics were prescribed as required and 0.12% chlorhexidine mouth rinse was recommended twice daily for one week. All implants followed a delayed loading protocol to allow undisturbed osseointegration. Patients were reviewed one week postoperatively for soft-tissue evaluation. At approximately six months healing abutments were replaced with compact conical abutments after clinical confirmation of implant stability. Three weeks later, the definitive prosthetic procedures started.

### 2.7. Prosthetic Rehabilitation

**Figure 3:**
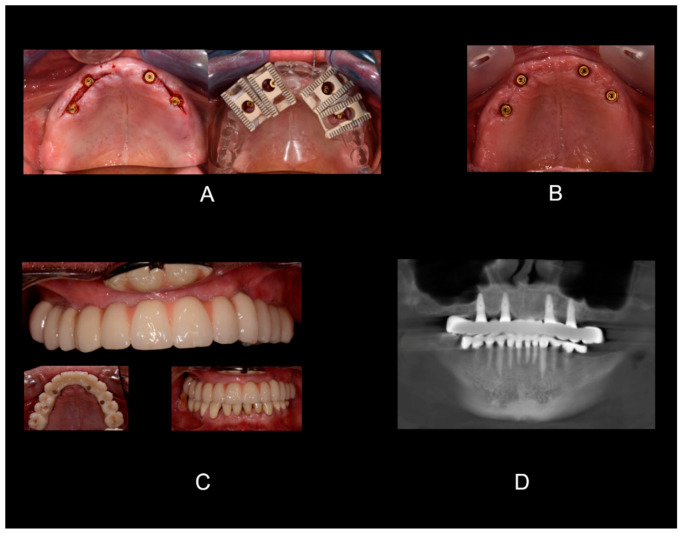
Prosthetic rehabilitation and postoperative follow-up. Sequential steps in the maxillary prosthetic phase following implant integration. (**A**) Placement of compact conical abutments on maxillary implants after a 6-month healing period, ensuring uniform emergence profile for prosthetic connection; (**B**) three-week postoperative control showing satisfactory soft tissue healing and abutment stability; (**C**) delivery of the definitive zirconia bridge on four maxillary implants with passive fit and functional occlusion; (**D**) one-year postoperative radiographic evaluation showing stable osseointegration and successful prosthetic integration.

Following successful healing and confirmation of implant stability, each patient received a screw-retained zirconia fixed bridge supported by four maxillary implants. The prosthesis was delivered after verifying passive fit, occlusion, phonetics, and esthetic parameters. The sequence of abutment placement, definitive restoration, and the one-year clinical follow-up is shown in Figure 3. Periapical radiographs obtained at the one-year visit were used exclusively for marginal bone loss evaluation in accordance with the ALADAIP principle (As Low As Diagnostically Acceptable being Indication-oriented and Patient-specific) [31].

### 2.8. Radiographic and Clinical Measurement

Patient-reported outcomes were evaluated using the validated Dutch version of the Oral Health Impact Profile-14 (OHIP-14) [32]. OHIP-14 scores were recorded at baseline defined as the situation with the existing maxillary denture prior to implant placement and at the one-year follow-up after delivery of the definitive prosthesis to quantify patient satisfaction. Radiographic evaluation of peri-implant marginal bone loss was performed using standardized periapical radiographs obtained at prosthetic baseline and at the 12-month follow-up using the paralleling technique with a Rinn positioning system and individualized bite registration at both time points. The radiographic reference landmark was the implant–abutment interface (implant shoulder); the distance from this landmark to the most coronal point of bone-to-implant contact was measured at the mesial and distal aspects of each implant. The known implant length was used as the per image calibration reference to control for magnification and films with detectable distortion of the implant outline were retaken. Because the reference point is prosthetic loading rather than implant placement, bone remodeling during the unloaded healing period is not captured by this protocol; this is acknowledged as a limitation. For each implant, mesial and distal bone level measurements were averaged to obtain a single implant-level marginal bone loss value, thereby preventing artificial inflation of the sample size by avoiding the treatment of two measurements from the same implant as independent observations [30]. Marginal bone loss was calculated as the difference in bone levels between time points. All radiographic measurements were performed by two independent examiners (M.T. and T.M.) using digital measurement software (ImageJ version 1.54f). Because implant surface (full-rough versus hybrid) is not radiographically distinguishable on standard periapical films and the allocation list was not consulted during measurement, blinding to surface allocation was effectively maintained; blinding to time point was not feasible because the prosthetic suprastructure is visible on the 12-month films. Intra-rater reliability was assessed by re-measurement of 10 randomly selected radiographs by M.T. and inter-rater reliability was assessed across the full dataset between M.T. and T.M. and both were quantified by intraclass correlation co-efficient (two-way mixed-effects, absolute-agreement, single-measurement). Consistent with the split-mouth design implant-level marginal bone loss values were compared between full-rough implants (IBT/IBNT/IBR) and hybrid-surface implants (MSC-IBT/MSC-IBNT). Probing depth was measured at six sites per implant at the one-year follow-up using a standardized periodontal probe (mesio-buccal, buccal, disto-buccal, mesio-palatal, palatal, disto-palatal), and the implant level mean in each patient was calculated as implants were clustered within the same patient.

### 2.9. Statistical Analysis

Two units of analysis were used. For the longitudinal baseline-to-12-month change, the unit of analysis was the implant; each implant contributed a paired pre/post-observation and the test is paired within implant so between-implant independence is not required. For the primary split-mouth surface comparison, the unit of analysis was the patient; within-patient mean change scores were calculated separately for full-rough and hybrid implants in each patient who contributed at least one of each surface with paired baseline + 12-month radiographs and the resulting paired (full-rough, hybrid) means were compared with a Wilcoxon signed-rank test, with the Hodges–Lehmann pseudo-median as the effect estimate. The same implant-level paired analysis was applied to absolute 12-month bone levels as a secondary surface comparison. Descriptive statistics (median and interquartile range) were calculated for all implant-level radiographic variables. Implant-level marginal bone loss was defined as the mean of mesial and distal measurements per implant. Longitudinal changes from prosthetic baseline to the 12-month follow-up were analyzed using paired Wilcoxon signed-rank testing at the implant level. Patients who received only one surface did not contribute to the surface comparison. A linear mixed effects sensitivity analysis was not performed because the number of paired patients does not justify the additional modeling assumptions. The surface comparison was not powered to detect equivalence or a pre-specified clinically meaningful difference. Probing depth measurements were summarized descriptively. OHIP-14 scores at the one-year follow-up were also summarized descriptively without inferential testing due to a pronounced floor effect (most patients scoring zero across most domains). A significance level of *p* < 0.05 was applied for all inferential comparisons. All statistical analyses were performed using R version 4.6.1 (R Foundation for Statistical Computing, Vienna, Austria).

## 3. Results

### 3.1. Study Completion and Follow-Up

All fifteen patients (seven males and eight females; mean age 62.5 ± 8.9 years) including three current smokers (P1, P8, P10) and 12 non-smokers were enrolled and underwent surgery. Sixty-one maxillary implants were placed. No intraoperative complications or postoperative infections were recorded. Four implants (6.6%) in three patients required removal and replacement during the observation period. There were three early failures (P3 tooth 12, removed at month 4 and replaced; P8 teeth 12 and 22, removed at month 2 and replaced two months later), all attributable to lack of osseointegration identified at the planned loading visit with no signs of infection, overload, or surgical trauma; there was one late failure (P13 tooth 22, removed at month ~26), recorded as progressive marginal bone loss without an identifiable mechanical or infectious precipitant. The per-patient early failure rate was 3/15 (20%) and the per-implant early failure rate was 3/61 (4.9%); the late failure occurred after the 12-month observation window. Three patients (P3, P6, P10) had their definitive restoration renewed during the observation period; in P3 and P6 the reason recorded was prosthesis fracture. One patient (P7) withdrew from clinical follow-up after surgery and is not included in the 12-month clinical or patient-reported outcomes; the remaining 14 patients attended the 12-month clinical visit. Paired baseline and 12-month radiographic data suitable for longitudinal analysis were available for 53 implants from 14 patients. The remaining 57 implants demonstrated stable clinical integration through the one-year follow-up.

### 3.2. Clinical Outcomes

Implant-level mean probing depths at the twelve-month follow-up (*n* = 14) ranged from 2 mm to 7 mm (median 4 mm); per-implant probing values were not recorded in this study, so implant-level probing statistics cannot be derived from this dataset. One patient (P12) had isolated bleeding spots on probing and detectable plaque on the implant collar at the 12-month visit; the remaining 13 patients followed clinically had no bleeding on probing recorded. No suppuration or mucosal dehiscence was observed in any patient.

### 3.3. Radiographic Outcomes

**Figure 4:**
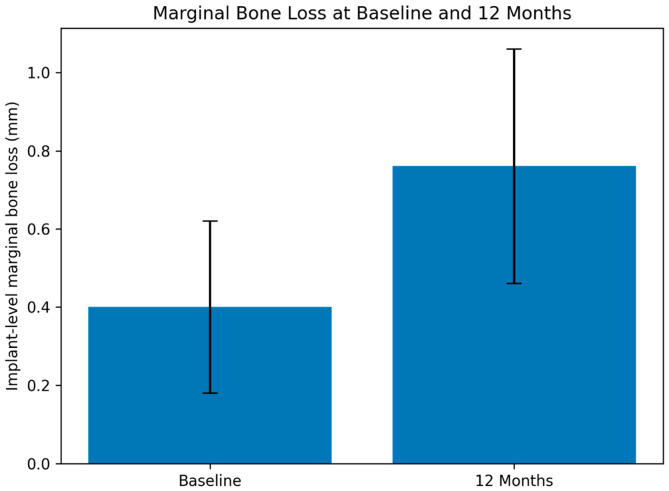
Implant-level paired marginal bone level at prosthetic baseline and at the 12-month follow-up for the 53 implants from 14 patients with paired data. Implant-level values were calculated as the mean of mesial and distal measurements per implant. The increase from baseline to 12 months was statistically significant (paired Wilcoxon signed-rank test, *p* < 0.001).

Paired baseline and 12-month radiographs suitable for longitudinal analysis were available for 53 implants from 14 patients. At prosthetic baseline the median implant-level marginal bone level was 0.38 mm (interquartile range [IQR] 0.20–0.54). At the 12-month follow-up the median had increased to 0.78 mm (IQR 0.47–1.32), corresponding to a median within-implant change of 0.28 mm (IQR 0.14–0.79) (paired Wilcoxon signed-rank test, *p* < 0.001; Figure 4). The distribution carried a long upper tail; the upper quartile reached 1.32 mm, and two implants (P2 tooth 22 and P14 tooth 15) exceeded 4 mm of 12-month bone loss. Both of these implants remained clinically stable, asymptomatic and free of suppuration at the follow-up visit but are noted because they approach or exceed thresholds commonly used as markers of progressive bone loss and warrant continued radiographic surveillance.

When disaggregated by surface type full-rough implants (*n* = 29 from 11 patients) had a median 12-month marginal bone level of 0.64 mm (IQR 0.41–0.78) and a median within-implant change of 0.22 mm (IQR 0.14–0.42). Hybrid implants (*n* = 18 from 9 patients) had a median 12-month bone level of 1.33 mm (IQR 0.56–1.78) and a median within-implant change of 0.81 mm (IQR 0.28–1.10). The primary split-mouth surface comparison was performed at the patient level on the eight patients (P2, P5, P6, P8, P9, P11, P12, P14) who contributed at least one full-rough and one hybrid implant with paired baseline and 12-month data. Within-patient mean change scores were lower for full-rough than for hybrid implants in every one of the eight pairs. The median within-patient paired difference (full-rough hybrid) was −0.49 mm, the Hodges–Lehmann pseudo-median was −0.55 mm, and the paired Wilcoxon signed-rank test gave *p* = 0.012. The corresponding patient-level paired comparison of absolute 12-month bone levels yielded a median paired difference of −0.38 mm (Hodges–Lehmann −0.41 mm; paired Wilcoxon *p* = 0.12). Patients with only one surface (P1, P4, P10) did not contribute to the surface comparison; their per-patient implant distribution is reported in Table 1. The direction of effect greater early bone change with hybrid than with full-rough implants is contrary to the prior expectation that a machined coronal collar would limit crestal remodeling; this finding should be interpreted in light of the small, unbalanced paired sample.

Site-specific descriptive measurements were generated to support the methodological choice of averaging mesial and distal values per implant. At the mesial aspect the median 12-month marginal bone level was 0.78 mm (IQR 0.44–1.65) and at the distal aspect it was 0.66 mm (IQR 0.38–1.28). An exploratory paired comparison between mesial and distal measurements within the same implant did not show a statistically significant difference (Wilcoxon signed-rank test, *p* = 0.17). Because mesial and distal measurements originate from the same implant and are not independent, all implant-level analyses used the within-implant mean of both sites as a single value in accordance with established methodological recommendations for split-mouth and within-implant repeated measures studies [26]. Both intra-rater (M.T., 10-radiograph remeasurement) and inter-rater (M.T. versus T.M., full dataset) reliability showed good agreement (ICC = 0.85).

### 3.4. Patient-Reported Outcomes

**Figure 5:**
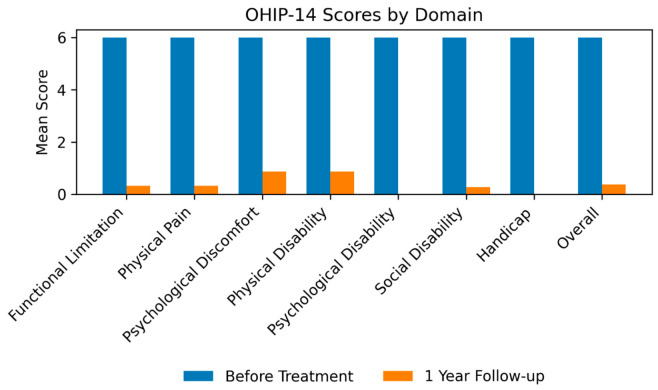
OHIP-14 scores at baseline and at the 12-month follow-up by domain. This distribution demonstrates a pronounced baseline ceiling effect and a follow-up floor effect, indicating excellent oral health-related quality of life following definitive maxillary rehabilitation.

At baseline all patients reported unsatisfactory oral health-related quality of life with their existing maxillary denture reflecting substantial functional, physical, and psychosocial impairment. At the one-year review (*n* = 14; one patient withdrew from clinical follow-up) scores decreased to near-zero levels across all items with follow-up means ranging from 0 to 0.87 indicating minimal or no perceived impact on oral health-related quality of life (Figure 5).

This pronounced change is consistent with resolution of the functional, physical, psychological, and social limitations present prior to treatment. Domains such as social disability and handicap reached a mean score of 0, while the remaining domains including physical pain and psychological discomfort showed only negligible residual values (<1).

Because of this marked floor effect and the extremely limited variance at follow-up no inferential statistical testing was performed. The floor effect is consistent with a strong treatment effect but may also reflect limited OHIP-14 sensitivity at the lower end of impairment; in patients moving from severe baseline impairment to a well-functioning fixed prosthesis or low baseline expectations the instrument cannot discriminate further within the near-floor region. These data are reported descriptively rather than as a graded patient-centered outcome.

## 4. Discussion

This pilot study evaluated the feasibility of adapting a prosthesis-based 3D-printed guide system for flapless multi-implant placement in the edentulous maxilla and assessed short-term marginal bone behavior in a split-mouth comparison of full-rough versus hybrid-surface implants.

The guide system developed and evaluated in this study builds upon the concept of prosthesis-based surgical guidance for implant placement extending a previously validated single-implant mandibular approach to the more demanding context of multi implant maxillary rehabilitation [19]. The incorporation of repositionable PEEK rails and interchangeable angulation correction sleeves addresses a common limitation of conventional printed guides, namely the inability to make intraoperative adjustments without fabricating an entirely new guide. This design philosophy is consistent with the broader principle that reduced infrastructure digital workflows should retain sufficient flexibility to accommodate the anatomical variability inherent to edentulous maxillae [12,14]. Guided flapless surgery in the edentulous maxilla has been shown to reduce operative time, preserve soft tissue architecture and limit postoperative morbidity compared with open flap approaches [33,34]. Mucosa-supported guides, the category to which the present system belongs, have demonstrated mean angular deviations of approximately 2.6–3.4° and mean shoulder deviations of approximately 0.9–1.2 mm in systematic reviews of fully edentulous maxillary cases [5,21]. The PEEK rail-and-sleeve system was not formally evaluated for accuracy in the present clinical study as no postoperative CBCT was obtained for deviation measurement; this is an acknowledged limitation. Nevertheless, the absence of intraoperative complications including sinus or nasal cavity encroachment or labial plate perforation and the attainment of adequate primary stability in 57 of 61 implants support clinical feasibility for this adapted design.

The per-implant early failure rate of 4.9% (three of 61 implants before loading with one additional late failure at approximately 26 months) falls within the range reported in the broader implant literature for the maxilla. Large-scale registry analyses have consistently identified the maxilla and particularly the anterior maxillary region as associated with higher failure rates than the mandible, with reported early failure rates in the range of 1.6–3.6% across mixed populations and higher in fully edentulous maxillary rehabilitation [35,36,37]. The higher failure incidence in this cohort is consistent with the challenges of the edentulous maxilla including reduced bone density and trabecular support [5,6]. Smoking was recorded in three of fifteen patients and all three early failures occurred in two of these three smokers corroborating the well-established association between tobacco use and impaired osseointegration. The delayed loading protocol adopted in this study with prosthetic loading deferred to approximately six months after placement is consistent with evidence that undisturbed healing remains the most predictable approach in the edentulous maxilla, particularly for bone of reduced density [38,39].

The median implant-level marginal bone level of 0.78 mm (IQR 0.47–1.32) at 12 months measured from prosthetic baseline is broadly consistent with data reported for implant-supported fixed prostheses in the edentulous maxilla. Long-term studies of fixed implant-supported maxillary rehabilitations have reported mean marginal bone loss below 1 mm over periods of 10 years or more with individual variation being considerable [10,40]. Immediately loaded full-arch maxillary protocols have reported 12-month marginal bone loss averaging approximately 0.9 mm comparable to the median observed in the present study [11]. The two implants with bone loss exceeding 4 mm at 12 months (P2 tooth 22 and P14 tooth 15) represent clinically significant outliers that approach or exceed thresholds used as markers of progressive peri-implant bone loss and warrant long-term surveillance even though both remained clinically asymptomatic. This distribution pattern showing a central tendency within physiological limits alongside a small number of high-loss outliers has been reported in other prospective studies and underscores the importance of monitoring individual implants rather than relying solely on group-level statistics [22,23].

The surface comparison constitutes the most biologically provocative finding of this pilot study. The a priori rationale for hybrid implants was that a machined coronal collar would limit early crestal biofilm accumulation and therefore reduce the magnitude of peri-implant bone remodeling in the critical early phase following prosthetic loading [28]. This expectation was not confirmed; full-rough implants showed lower within-patient mean marginal bone change at 12 months than hybrid implants in all eight evaluable paired patients with a Hodges–Lehmann pseudo-median difference of −0.55 mm (*p* = 0.012). This finding contradicts the premise that a machined coronal collar is bone protective in the short term. The literature on this question is not settled. A 2016 systematic review and meta-analysis including 12 studies found less marginal bone loss at rough-surfaced and rough-surfaced micro-threaded collar implants compared with machined neck implants (difference in means 0.321 mm, 95% CI 0.149–0.493) suggesting that roughened coronal surfaces may confer an advantage for early bone preservation through enhanced bone-to-implant contact [41]. More recently a three-year randomized controlled trial in periodontitis-susceptible patients found comparable marginal bone level changes between hybrid and full-rough implants (mean change −0.08 versus 0.02 mm) suggesting functional equivalence under defined conditions [19]. A 4-year split-mouth randomized controlled trial on immediately loaded full-arch maxillary implants which directly parallels the design of the present study found no significant difference in crestal bone levels between hybrid and roughened implants with similar plaque and bleeding scores [28]. The discordance between the present pilot findings and these more controlled datasets may reflect several non-surface factors including unbalanced per-patient implant numbers (the hybrid side had fewer implants per-patient in several cases), possible differences in tissue biotype or prosthetic loading distribution between sides, and the non-randomized side allocation. A further consideration is implant positioning depth; if hybrid implants were placed with the machined collar at the subcrestal level rather than at the crestal level, the functional roughened surface may have been located more coronally relative to the peri-implant soft tissue seal which has been associated with more crestal remodeling [25]. These factors cannot be disentangled with the available dataset reinforcing the conclusion that the surface comparison should be regarded as hypothesis-generating rather than definitive. The Glibert et al. split-mouth studies from the same research group, using a more balanced design and longer follow-up, provide stronger evidence regarding the long-term surface effect and should be referenced alongside the present preliminary data [36,37].

Patient-reported outcomes measured by the OHIP-14 at one year showed a pronounced floor effect with most patients scoring near zero across all domains. This pattern is highly consistent with the existing literature on implant-supported fixed prostheses in edentulous patients. Studies have consistently demonstrated that conversion from a conventional removable complete denture to an implant-supported fixed prosthesis produces large and sustained improvements in oral health-related quality of life, with gains in functional, psychological, and social domains [32]. The floor effect observed at one year in this cohort reflects not a limitation of the intervention but the degree to which the severe baseline impairment was resolved following definitive maxillary rehabilitation. Comparable OHIP-14 floor effects at 12 months have been reported following immediately loaded implant-supported prostheses in the edentulous maxilla, with quality-of-life stabilization observed at approximately one year after surgery [31]. The finding that the OHIP-14 lacks sufficient resolution to discriminate between degrees of excellent oral health in patients who have transitioned from severe denture-associated impairment to a well-functioning fixed prosthesis is a recognized measurement limitation of the instrument in this clinical context. Future studies in this population might benefit from supplementary patient-reported outcome measures with greater sensitivity at the lower end of the impairment spectrum, or from qualitative assessments of patient experience.

Several limitations of the present study warrant careful consideration. First, the sample size of 15 patients with only eight contributing paired data to the surface comparison is insufficient to exclude a clinically meaningful surface effect in either direction; statistical power was not a design objective of this feasibility pilot. Second, the non-randomized side allocation with hybrid implants assigned by alternating sequence and three patients receiving only full-rough implants for prosthetic reasons introduces potential selection bias that cannot be adjusted post hoc. Third, the radiographic baseline was defined as prosthetic loading at six months rather than at implant placement meaning that bone remodeling occurring during the unloaded healing phase is not captured; the reported marginal bone change therefore underestimates total bone remodeling from placement to 12 months. Fourth, no postoperative CBCT was obtained to quantify angular or linear deviations from the virtual plan so the accuracy of the guide system cannot be formally characterized from this dataset. Despite these limitations, the study contributes clinically relevant feasibility data on an adaptable prosthesis-based guide system for maxillary flapless implant rehabilitation and identifies a potentially unexpected surface-related bone remodeling signal that merits investigation in adequately powered formally randomized trials.

## 5. Conclusions

This prospective pilot study demonstrates that a prosthesis-based 3D-printed guide system, incorporating repositionable PEEK rails and interchangeable angulation correction sleeves, can be feasibly adapted for flapless multi-implant placement in the edentulous maxilla. Overall implant-level marginal bone loss from prosthetic baseline to 12 months was within the range commonly reported as physiological early crestal remodeling and patient-reported oral health-related quality of life improved markedly following definitive prosthetic rehabilitation. Contrary to the working hypothesis, hybrid-surface implants showed greater early marginal bone change than full-rough implants. The prosthesis-based guide system represents a reduced infrastructure alternative to proprietary guided-surgery platforms; however, it remains operator-dependent and requires CBCT imaging and access to a desktop 3D printer and is therefore best characterized as a reduced-infrastructure rather than a low-infrastructure approach.

## Figures and Tables

**Table 1:** Distribution of implants placed in the maxilla.

Implant System	Surface Type	Diameter (mm)	Length (mm)	Number of Implants, *n*
IBT	Full-rough	4.0	8.5	2
IBT	Full-rough	4.0	10.0	20
IBT	Full-rough	4.0	11.5	4
IBNT	Full-rough	3.25	8.5	1
IBNT	Full-rough	3.25	10.0	7
IBNT	Full-rough	3.25	11.5	1
IBR	Full-rough	4.0	8.5	1
MSC-IBT	Hybrid	4.0	8.5	1
MSC-IBT	Hybrid	4.0	10.0	13
MSC-IBT	Hybrid	4.0	11.5	2
MSC-IBNT	Hybrid	3.25	8.5	1
MSC-IBNT	Hybrid	3.25	10.0	7
MSC-IBNT	Hybrid	3.25	11.5	1

Distribution of maxillary implants by system, surface, diameter, and length (*n* = 61 in 15 patients). Full-rough implants (IBT, IBNT, IBR) are roughened along their entire length. Hybrid implants (MSC-IBT, MSC-IBNT) have a rough apical and middle third with a machined smooth coronal collar. IBT = 4.0 mm diameter; IBNT = 3.25 mm diameter; IBR = 4.0 mm full-rough variant.

## Data Availability

The data presented in this study are available on request from the corresponding author.

## References

[1] Nascimento G.G., Alves-Costa S., Romandini M. (2024). Burden of Severe Periodontitis and Edentulism in 2021, with Projections up to 2050: The Global Burden of Disease 2021 Study. J. Periodontal Res..

[2] Carlsson G.E., Omar R. (2010). The Future of Complete Dentures in Oral Rehabilitation. A Critical Review. J. Oral Rehabil..

[3] Emami E., de Souza R.F., Kabawat M., Feine J.S. (2013). The Impact of Edentulism on Oral and General Health. Int. J. Dent..

[4] Felton D.A. (2016). Complete Edentulism and Comorbid Diseases: An Update. J. Prosthodont..

[5] Jaffin R.A., Berman C.L. (1991). The Excessive Loss of Branemark Fixtures in Type IV Bone: A 5-Year Analysis. J. Periodontol..

[6] Mericske-Stern R., Taylor T.D., Belser U. (2000). Management of the Edentulous Maxilla with Implant-Supported Prostheses. Clin. Oral Implant. Res..

[7] Gonzalez-Gonzalez I., Dellanos-Lanchares H., Brizuela-Velasco A., Alvarez-Riesgo J.A., Llorente-Pendas S., Herrero-Climent M., Alvarez-Arenal A. (2020). Complications of Fixed Full-Arch Implant-Supported Metal-Ceramic Prostheses. Int. J. Environ. Res. Public Health.

[8] Zarb G.A., Schmitt A. (1996). The Edentulous Predicament. II: The Longitudinal Effectiveness of Implant-Supported Overdentures. J. Am. Dent. Assoc..

[9] Slot W., Raghoebar G.M., Vissink A., Huddleston Slater J.J., Meijer H.J.A. (2010). A Systematic Review of Implant-Supported Maxillary Overdentures after a Mean Observation Period of at Least 1 Year. J. Clin. Periodontol..

[10] De Bruyn H., Collaert B., Linden U., Björnsson S., Gustafsson T. (1997). Patient Satisfaction with Turned Titanium Implants Placed in the Edentulous Mandible. Clin. Oral Implant. Res..

[11] Bryant S.R. (1998). The Effects of Age, Jaw Site, and Bone Condition on Oral Implant Outcomes. Int. J. Prosthodont..

[12] Vercruyssen M., Laleman I., Jacobs R., Quirynen M. (2015). Computer-Supported Implant Planning and Guided Surgery: A Narrative Review. Clin. Oral Implant. Res..

[13] Bornstein M.M., Scarfe W.C., Vaughn V.M., Jacobs R. (2014). Cone Beam Computed Tomography in Implant Dentistry: A Systematic Review. Int. J. Oral Maxillofac. Implant..

[14] Schneider D., Marquardt P., Zwahlen M., Jung R.E. (2009). A Systematic Review on the Accuracy and the Clinical Outcome of Computer-Guided Template-Based Implant Dentistry. Clin. Oral Implant. Res..

[15] D’haese J., Ackhurst J., Wismeijer D., De Bruyn H., Tahmaseb A. (2017). Current State of the Art of Computer-Guided Implant Surgery. Periodontol. 2000.

[16] Valente F., Schiroli G., Sbrenna A. (2009). Accuracy of Computer-Aided Oral Implant Surgery. Int. J. Oral Maxillofac. Implant..

[17] Cassetta M., Stefanelli L.V., Giansanti M., Di Mambro A., Calasso S. (2011). Depth Deviation and Occurrence of Early Surgical Complications Using a Single Stereolithographic Guide. Int. J. Oral Maxillofac. Surg..

[18] Tudts M., D’haese R., Hommez G., Christiaens V., Vandeweghe S. (2024). Proof of Concept of a New 3D-Guided System for a Single Implant Overdenture in the Mandible: An In Vitro Study. Int. J. Oral Maxillofac. Implant..

[19] D’haese J., Van De Velde T., Elaut L., De Bruyn H. (2012). A Prospective Study on the Accuracy of Mucosally Supported Stereolithographic Surgical Guides in Fully Edentulous Maxillae. Clin. Implant. Dent. Relat. Res..

[20] Tahmaseb A., Wu V., Wismeijer D., Coucke W., Evans C. (2014). The Accuracy of Computer-Guided Implant Surgery with Mucosa-Supported Surgical Templates in the Edentulous Maxilla: A Systematic Review. Clin. Oral Implant. Res..

[21] Jokstad A., Sanz M., Palmer P., Renouard F. (2009). Consensus Statements and Recommended Clinical Procedures Regarding Computer-Assisted Implant Dentistry. Int. J. Oral Maxillofac. Implant..

[22] Berglundh T., Armitage G., Araujo M.G., Avila-Ortiz G., Blanco J., Camargo P.M., Chen S., Cochran D., Derks J., Figuero E. (2018). Peri-Implant Diseases and Conditions: Consensus Report of Workgroup 4 of the 2017 World Workshop on the Classification of Periodontal and Peri-Implant Diseases and Conditions. J. Clin. Periodontol..

[23] Schwarz F., Derks J., Monje A., Wang H.-L. (2018). Peri-Implantitis. J. Clin. Periodontol..

[24] Albrektsson T., Wennerberg A. (2004). Oral Implant Surfaces: Part 1—Review Focusing on Topographic and Chemical Properties of Different Surfaces and In Vivo Responses to Them. Int. J. Prosthodont..

[25] De Bruyn H., Pivovarova M., Rondas A., Scheldeman M., Op de Laak H., Vandeweghe S. (2025). Survival and Bone Remodeling in Hybrid Surface Dental Implants Placed with 3 Surgical Protocols up to 5 Years: A Retrospective Practice-Based Cohort Study. J. Clin. Med..

[26] Koodaryan R., Hafezeqoran A. (2016). Evaluation of Implant Collar Surfaces for Marginal Bone Loss: A Systematic Review and Meta-Analysis. BioMed Res. Int..

[27] Serrano J., Montero E., Llancapal-Vergara M., Alonso B., Sanz M., Herrera D. (2025). Three-Year Outcomes of Dental Implants with a Hybrid Surface Macro-Design Placed in Patients with History of Periodontitis: A Randomised Clinical Trial. J. Clin. Periodontol..

[28] Matthijs S., Christiaens V., Matthys C., De Bruyn H., Glibert M. (2025). A 6-Year Randomized Controlled Trial on Different Implant Designs in Maxillary Overdenture Patients. Clin. Implant. Dent. Relat. Res..

[29] Offord D., Kingsford N., Glibert M., Pitman J., Christiaens V. (2025). Four-Year Outcomes of Immediately Loaded Full-Arch Maxillary Dental Implants with Hybrid Versus Roughened Surfaces: A Split-Mouth Randomized Controlled Trial. J. Clin. Med..

[30] Glibert M., Vervaeke S., Ibrahim W., Doornewaard R., De Bruyn H. (2023). A Split-Mouth Study to Assess the Effect of Implant Surface Roughness on Implant Treatment Outcome After 5 Years. Int. J. Periodontics Restor. Dent..

[31] Glibert M., Matthys C., Van Lancker A., Segers A., De Bruyn H. (2024). A Long-Term Split-Mouth Randomized Controlled Trial to Assess Implant Treatment Outcome Using Implants with a Different Surface Roughness. Appl. Sci..

[32] Lesaffre E., Garcia Zattera M.-J., Redmond C., Huber H., Needleman I., ISCB Subcommittee on Dentistry (2007). Reported Methodological Quality of Split-Mouth Studies. J. Clin. Periodontol..

[33] de Mendonça R.P., Estrela C., Bueno M.R., Carvalho T.C.A.S.G., Estrela L.R.d.A., Chilvarquer I. (2025). Principles of Radiological Protection and Application of ALARA, ALADA, and ALADAIP: A Critical Review. Braz. Oral Res..

[34] van der Meulen M.J., John M.T., Naeije M., Lobbezoo F. (2008). The Dutch Version of the Oral Health Impact Profile (OHIP-NL): Translation, Reliability and Construct Validity. BMC Oral Health.

[35] Jeong S.M., Choi B.H., Li J., Kim H.S., Ko C.Y., Jung J.H. (2007). Flapless Implant Surgery: An Experimental Study. Oral Surg. Oral Med. Oral Pathol. Oral Radiol. Endod..

[36] Campelo L.D., Camara J.R.D. (2002). Flapless Implant Surgery: A 10-Year Clinical Retrospective Analysis. Int. J. Oral Maxillofac. Implant..

[37] Comyn P.A., Laverty D.P., Buglass J., Patel A. (2018). Flapless Dental Implant Surgery and Use of Cone Beam Computer Tomography Guided Surgery. Br. Dent. J..

[38] Chrcanovic B.R., Albrektsson T., Wennerberg A. (2015). Smoking and Dental Implants: A Systematic Review and Meta-Analysis. J. Dent..

[39] Esposito M., Grusovin M.G., Maghaireh H., Worthington H.V. (2013). Interventions for Replacing Missing Teeth: Different Times for Loading Dental Implants. Cochrane Database Syst. Rev..

[40] Maló P., de Araújo Nobre M., Lopes A., Francischone C., Rigolizzo M. (2012). “All-on-4” Immediate-Function Concept for Completely Edentulous Maxillae: A Clinical Report on the Medium (3 Years) and Long-Term (5 Years) Outcomes. Clin. Implant. Dent. Relat. Res..

[41] Vanden Bogaerde L., Rangert B., Wendelhag I. (2005). Immediate/Early Function of Brånemark System TiUnite Implants in Fresh Extraction Sockets. Clin. Implant. Dent. Relat. Res..

